# Step Count as a Digital Mobility Outcome—A Pilot Study on Differences Between the Upper and Lower Extremities

**DOI:** 10.3390/s26154843

**Published:** 2026-08-01

**Authors:** Dannik Haas, Carolina Vogel, Chiara N. Meierhofer, Kira Hofmann, Tanja C. Maisenbacher, Maximilian M. Menger, Steven C. Herath, Tina Histing, Benedikt J. Braun

**Affiliations:** Department of Trauma and Reconstructive Surgery, Eberhard-Karls-University Tuebingen, BG Klinik Tuebingen, Schnarrenbergstr. 95, 72076 Tuebingen, Germany; cvogel@bgu-tuebingen.de (C.V.); tmaisenbacher@bgu-tuebingen.de (T.C.M.); mmenger@bgu-tuebingen.de (M.M.M.); sherath@bgu-tuebingen.de (S.C.H.); thisting@bgu-tuebingen.de (T.H.); bbraun@bgu-tuebingen.de (B.J.B.)

**Keywords:** wearables, physical activity, digital monitoring, rehabilitation, objective outcome

## Abstract

Background: Fractures of the lower extremity are commonly associated with impaired mobility, whereas injuries of the upper extremity primarily affect limb-specific function. Wearable devices allow the objective and continuous monitoring of physical activity and may therefore provide additional insight into postoperative recovery processes under real-world conditions. This exploratory study aimed to compare postoperative trajectories of daily step count following surgical treatment of upper versus lower extremity fractures and to explore possible associations with general health status and return to work. Methods: In this prospective observational study, 38 patients with upper extremity fractures and 55 patients with lower extremity fractures were included. Daily step counts were recorded postoperatively. Patient-reported general health (PROMIS Global Health) and work status at three months were assessed. Results: During the early postoperative period, patients with lower extremity injuries recorded lower daily step counts than patients with upper extremity injuries. Within three months, 89% of patients with upper extremity injuries and 49% of patients with lower extremity injuries had returned to work. Conclusions: In this exploratory cohort, postoperative step count trajectories differed between surgically treated upper and lower extremity fractures during the early postoperative period. Return-to-work rates at three months also differed between groups. Interpretation of these findings is limited by the real-world use of heterogeneous consumer wearable devices and the exploratory study design. These findings should be considered preliminary and hypothesis-generating.

## 1. Introduction

Musculoskeletal injuries of the extremities are among the most frequent causes of temporary or permanent functional impairment and contribute substantially to reduced quality of life and socioeconomic burden [1]. Fractures of both the upper and lower extremities can impair independence, daily activity, and work ability [2,3], making restoration of function a primary goal of trauma rehabilitation [4].

Postoperative recovery is traditionally assessed using clinical examination, imaging, and patient-reported outcome measures (PROMs). While PROMs such as the PROMIS Global Health Score provide valuable insight into perceived health status, they are subjective and do not allow continuous assessment of actual activity behavior in daily life [5,6]. Wearable devices enable objective and longitudinal monitoring of physical activity under real-world conditions. In recent years, the use of wearable sensors has gained increasing importance across various medical disciplines [7,8,9,10]. These devices—e.g., wristbands, smartwatches, or fitness trackers—enable the recording of objective activity parameters such as step count, movement duration, or energy expenditure [11]. A key advantage is that wearables have become widely available and are commonly used in everyday life, even among older adults [12,13]. This widespread availability enables clinical research under real-life conditions without the need for invasive or costly measurement techniques [14,15]. Previous research in musculoskeletal rehabilitation has primarily focused on arthroplasty [16,17,18,19]. However, previous studies have also demonstrated that wearable-based monitoring is applicable in the context of acute musculoskeletal trauma. In orthopedic trauma research, daily step count represents the most frequently used digital outcome parameter [20,21], as it can be reliably recorded by most wearable devices [21,22].

In our previous studies, a marked reduction in step count immediately following lower extremity surgery with gradual recovery during rehabilitation has been demonstrated, showing an association with clinical outcomes such as pain reduction and return to work [23,24]. In contrast, data on activity development following upper extremity injuries remain limited. While walking ability is typically preserved, previous observations suggest that step count may still reflect changes in overall activity behavior in this population [23].

Importantly, injuries of the upper and lower extremities differ fundamentally in their biomechanical and functional consequences. Comparing both injury locations allows evaluation of step count trajectories within a shared digital mobility metric and highlights context-dependent interpretation across different functional conditions.

The objective of this exploratory study was to compare postoperative step count trajectories following surgical treatment of upper and lower extremity fractures. In addition, exploratory associations between early postoperative step count, patient-reported general health, and return to work at three months were examined to assess the potential role and limitations of step count as an objective digital mobility outcome. Where available, pre-injury step count was documented to contextualize postoperative trajectories.

## 2. Materials and Methods

This exploratory observational study was conducted as a prospective clinical investigation. Selected clinical parameters were supplemented retrospectively.

A total of 93 adult patients were included: 38 with surgically treated upper extremity fractures and 55 with lower extremity fractures. All patients were treated at a tertiary care center.

Inclusion criteria were age ≥18 years, surgical treatment of at least one upper or lower extremity fracture, ownership and habitual use of a personal wearable device prior to injury, and ability to provide informed consent. No age-based exclusion for retirement status was applied.

Exclusion criteria included current or prior substance abuse, pregnancy or planned pregnancy during the study period, incarceration, and participation in another clinical trial with potential influence on outcomes.

Participants used their personal wearable devices under real-life conditions according to their usual daily habits. No standardized protocol regarding device wear (e.g., minimum daily wear time, wear location, or activity-specific use) was imposed in order to preserve habitual real-world behavior. All consumer wearable devices capable of recording daily step count were accepted. Device manufacturer was shared by the patients (optional). Software version, wear location, and firmware information were not recorded. Consequently, step count measurements reflect real-world device-derived activity obtained from heterogeneous consumer ecosystems rather than a standardized assessment of ambulatory activity. Step count data were manually extracted from each participant’s device in a standardized manner. Pre-injury step counts were extracted from wearable device records covering a defined baseline period prior to injury, where available. Because participants used their personal devices without supervision, actual daily wear time could not be verified retrospectively. The number of days with available postoperative data was assessed.

### 2.1. Data Collection and Outcomes

Recorded variables included age, sex, injury location (upper vs. lower extremity), PROMIS Global Health Score, and work status at three months postoperatively. If available, average daily step count prior to injury was documented. Occupational category, physical job demands, employment characteristics (e.g., self-employment or workplace accommodations), fracture severity, postoperative weight-bearing restrictions, rehabilitation protocols, and other detailed treatment characteristics were not systematically recorded in this exploratory analysis.

The primary outcome was postoperative daily step count recorded during routine follow-up for up to approximately 100 postoperative days. Secondary outcomes were PROMIS Global Health Score and return to work at three months. A daily step count threshold of 5000 steps was analyzed descriptively as a pragmatic activity milestone. At present, there is no universally accepted evidence-based definition of a specific step count reflecting adequate recovery or health across patient populations. The selected threshold therefore served as a comparative reference point rather than a normative or clinical benchmark.

### 2.2. Statistical Analysis and Ethics

Descriptive analyses were performed for the entire cohort and stratified by injury location. Given the exploratory character of the study and the heterogeneous real-world data acquisition, longitudinal step count trajectories were analyzed descriptively. No formal repeated-measures model was performed because of the exploratory nature of the study. Logistic regression analyses were performed to investigate potential associations between early postoperative step counts (first two weeks) and subsequent PROM outcome as well as return to work at three months. These analyses were intended for hypothesis generation rather than prediction model development. Normality of continuous variables was assessed using the Shapiro–Wilk test. Between-group comparisons were performed using an independent-samples *t*-test for normally distributed data or the Mann–Whitney U test otherwise. Pre-injury mean daily step count, where available, and time to first achieve 5000 steps/day were compared between groups accordingly. Differences in return-to-work frequency at three months were assessed using a chi-square test with Yates correction, with Fisher’s exact test used as a sensitivity analysis. An alpha level of 0.05 was used for normality testing.

Statistical analyses and graphical visualizations were performed using JASP (Version 0.95.4), Jamovi (Version 2.6.26.0) and GraphPad Prism (Version 8.0.1). A positive ethics committee approval was obtained (Protocol code 790/2020BO2, approval date 18 November 2020). All participants provided written informed consent.

## 3. Results

Of the 93 included patients, 38 (41%) sustained upper extremity injuries and 55 (59%) had a lower extremity injury. Pre-injury step count data were available for 25 of 38 patients in the upper extremity group and for 44 of 55 patients in the lower extremity group. Accordingly, all descriptive comparisons including pre-injury step counts are based on the available subset of participants. Where available, pre-injury mean daily step counts did not differ between groups (Mann–Whitney U = 544, *p* = 0.945). Normality testing of pre-injury mean daily step counts using the Shapiro–Wilk test yielded *p* = 0.051 in the upper extremity group and *p* = 0.017 in the lower extremity group. Where available, mean pre-injury daily step count was 5866 (SD 2072) steps/day in the upper extremity group and 6365 (SD 2994) steps/day in the lower extremity group.

**Figure 1:**
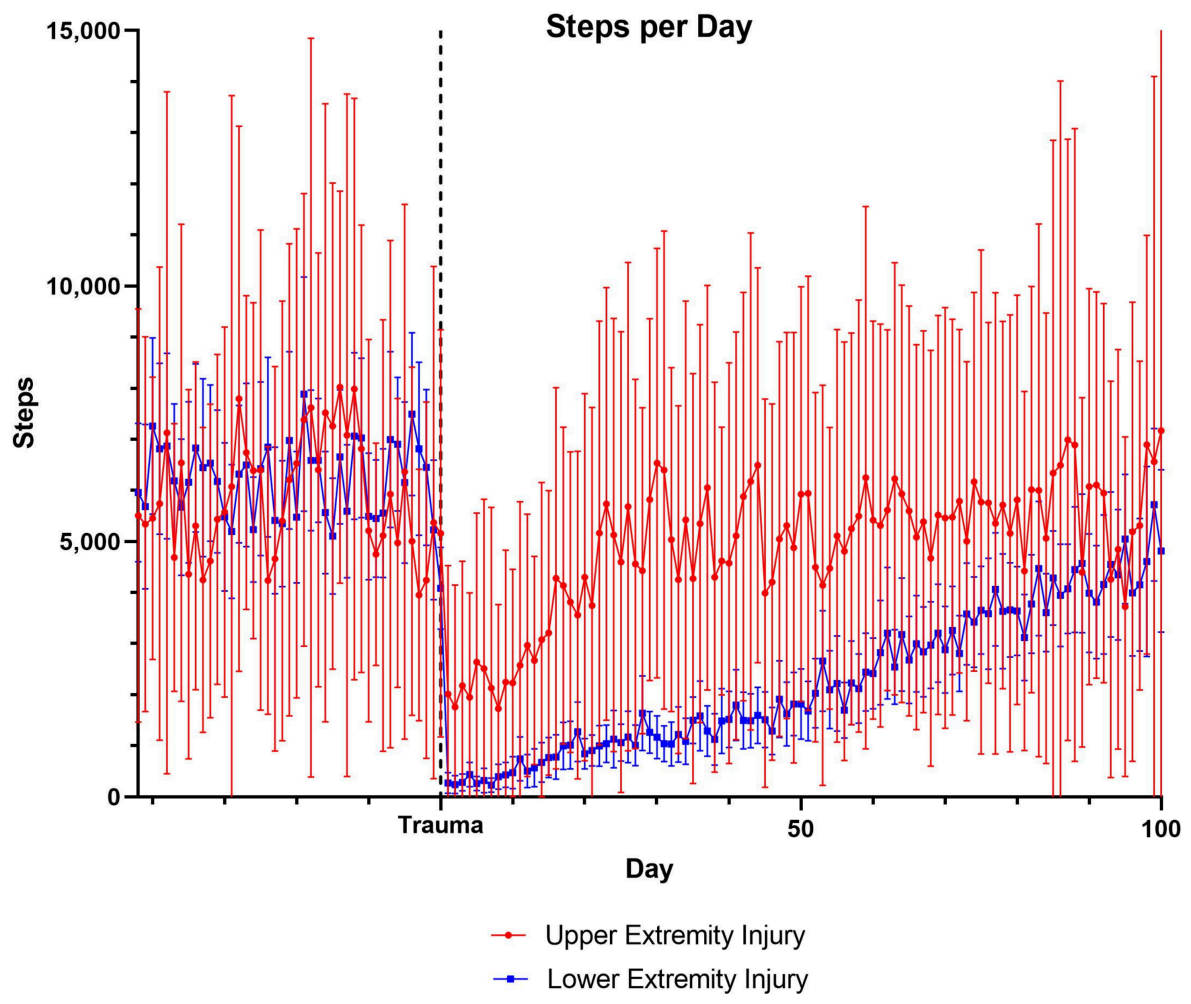
The graph shows the average daily step count from pre-injury day 42 (where available) to postoperative day 100 for patients with an upper (red dots) and lower (blue squares) extremity injury. The lines delineate the 95% confidence interval.

The comparison of mean steps per day is presented in Figure 1. During the early postoperative period, patients with lower extremity fractures recorded lower daily step counts than patients with upper extremity fractures. Step counts increased over time in both groups, and mean step counts converged between groups. Because monitoring duration varied between participants, the number of observations contributing to the daily means was not constant throughout follow-up. On average, data was available on 88.3% of postoperative days.

Among all participants, 78% provided information regarding their tracking hardware, while 22% declined to share their device type. The cohort was heavily dominated by two manufacturers: Apple devices were the most prevalent, utilized by 36%, followed closely by Samsung devices (20.5%). The remaining participants relied on a diverse mix of other commercial activity trackers and platforms, including Google (7%), Huawei (7%), Garmin (3%), standalone pedometers (2%), and Huawei Health, Polar, and Withings devices (1% each). Statistical analysis showed no significant association between the choice of wearable device and injury location. There were no statistically significant differences in clinical outcomes between the two primary device cohorts (Apple vs. Samsung) regarding pain scores, PROM, or return-to-work rates.

Furthermore, the study investigated whether and when patients first achieved 5000 steps in a single day during the observation period. Time to first achieve 5000 steps/day differed descriptively between groups among patients who achieved the threshold (Mann–Whitney U = 203, *p* = <0.05). Within the observation period, 95% of patients with upper extremity injuries achieved at least one day with 5000 steps, compared to 73% of patients with lower extremity injuries. Participants who did not reach the threshold during follow-up were not included in this comparison. The mean time to reach this threshold was 19 days for upper extremity injuries and 58 days for lower extremity injuries. Considering the median values, this difference became even more pronounced (13 days vs. 59 days).

**Figure 2:**
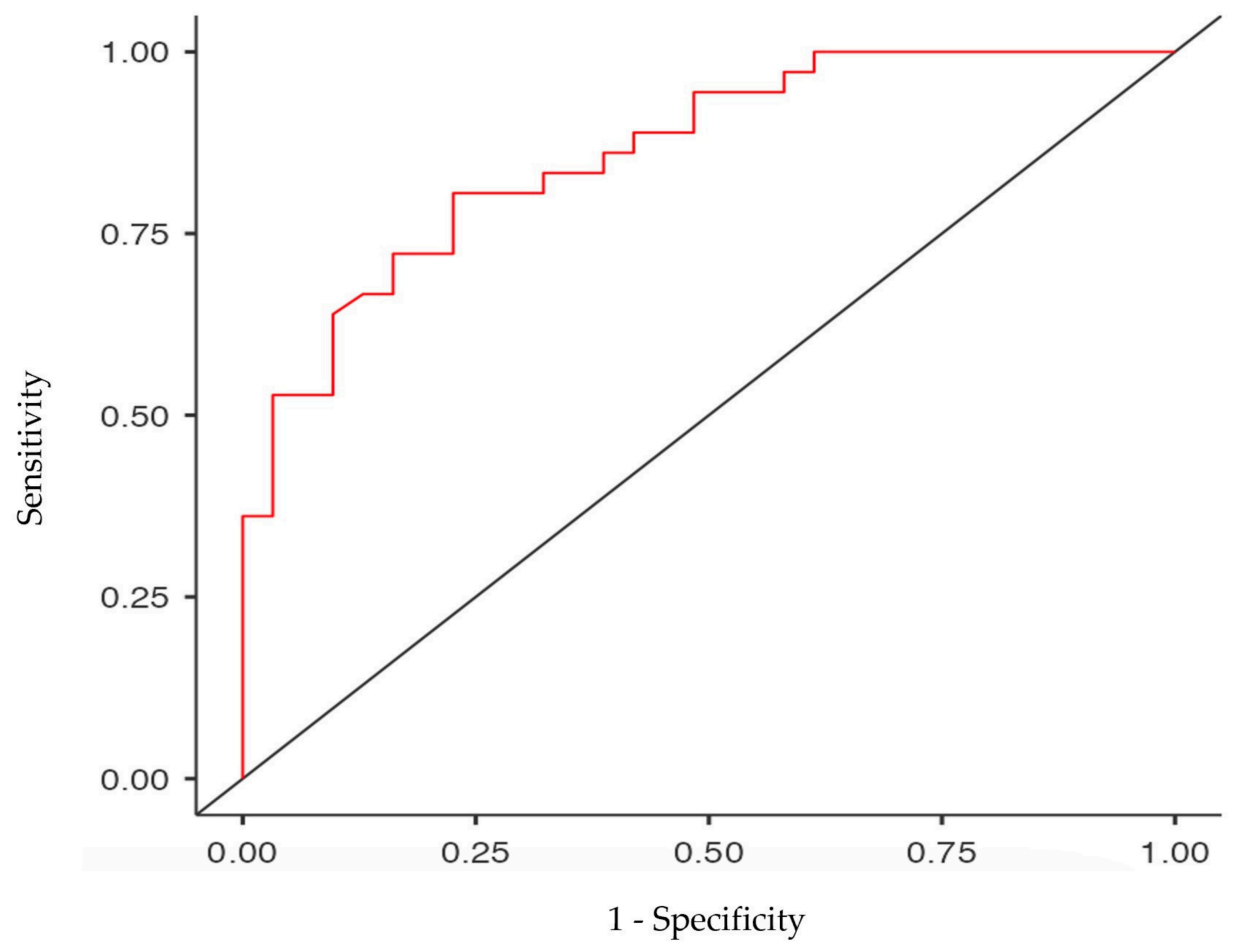
ROC Curve for Logistic Regression Model Predicting Patients’ PROM outcome in the top half.

**Figure 3:**
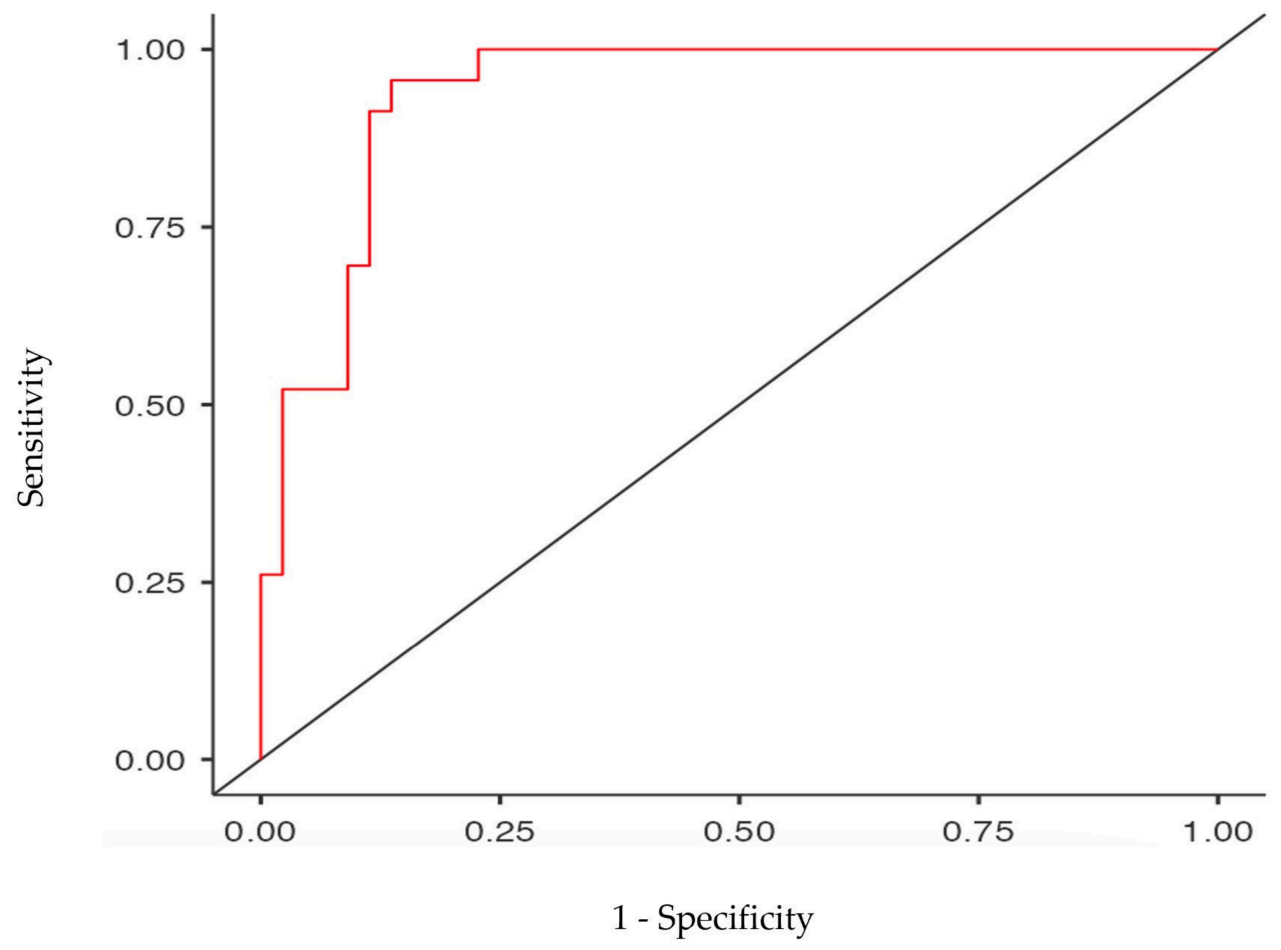
ROC Curve for the Logistic Regression Model Predicting Patients’ return to work status.

At three months postoperatively, 89% of patients with upper extremity injuries (34/38) and 49% of patients with lower extremity injuries (27/55) had returned to work (chi-square test with Yates correction, χ^2^(1) = 14.50, *p* = 0.00014, Fisher’s exact *p* = <0.05). To explore the association between early postoperative activity and clinical outcomes, exploratory logistic regression analyses were performed. These analyses were intended for hypothesis generation and should not be interpreted as clinically validated prediction models. Model discrimination within the present dataset was assessed using ROC curves and summarized by the area under the curve (AUC). For the overall model including all patients, the AUC was 0.862 for PROM outcome (Figure 2) and 0.938 for return to work (Figure 3).

When splitting the predictive model for upper and lower extremity patients, the capacity for the lower extremity remained the same as above, whereas a complete separation was observed in the upper extremity subgroup. Here, only 4 patients did not return to work, precluding meaningful subgroup analysis because of the very small number of non-events. These subgroup analyses should therefore be interpreted with considerable caution.

## 4. Discussion

This exploratory observational study examined postoperative trajectories of daily step count following surgically treated upper and lower extremity fractures and evaluated their association with patient-reported health and return to work. The primary objective was to compare postoperative step count trajectories between upper and lower extremity fractures and to explore their association with patient-reported outcomes and return to work.

The results show lower recorded step counts in patients with lower extremity injuries than in those with upper extremity injuries during the early postoperative period. In this context, step count captures ambulatory activity and general mobility and is therefore more directly related to mobility limitations in lower extremity injuries. It should therefore be interpreted as a pragmatic indicator of overall ambulatory activity rather than a comprehensive measure of functional recovery. The observed reduction in daily step count among patients with lower extremity injuries is consistent with expected functional limitations after lower limb trauma. Compared to upper extremity injuries, lower extremity injuries are typically associated with temporary or longer-term limitations in mobility. Postoperative partial weight-bearing, immobilization, or the use of walking aids inevitably lead to a significant reduction in daily activity and, consequently, in step count. In contrast, gait ability is largely preserved in patients with upper extremity injuries. Although pain, compensatory postures, or psychological factors may also lead to a slight decrease in activity, the restriction is generally smaller in magnitude than in lower extremity injuries [25,26]. These differences highlight that step count primarily captures ambulatory activity and therefore reflects different aspects of recovery depending on injury location. Furthermore, the interpretation of recorded step counts should consider the heterogeneity of consumer wearable devices used in this study. Previous systematic reviews have generally reported favorable validity and reliability for step count measurements from consumer wearable devices, while also highlighting variability in measurement performance across devices and manufacturers [27,28]. Such variability may arise from differences in proprietary step-detection algorithms and wear locations, while limited transparency regarding the underlying algorithms further complicates comparisons across consumer wearable devices [29]. In particular, wrist-worn devices may underestimate walking activity in patients with upper extremity immobilization because reduced arm swing can impair step detection. Consequently, the present findings reflect device-derived step counts obtained under everyday conditions rather than a standardized measurement of ambulatory activity. However, postoperative step count data availability was high (over 80%), highlighting the feasibility of this pragmatic approach for collecting longitudinal mobility data under real-world conditions. Although device information was available only for a subset of participants, exploratory analyses did not reveal an association between the predominant wearable device types and injury location. Likewise, no statistically significant differences in pain, PROM outcomes, or return-to-work rates were observed between the two largest device groups (Apple and Samsung). Nevertheless, these findings should be interpreted cautiously because device information remained incomplete and the smaller device groups were underrepresented. Therefore, these exploratory analyses reduce, but do not eliminate, the possibility of device-related measurement bias. Accordingly, temporal changes in step count may serve as a supportive, activity-based indicator of postoperative recovery in lower extremity injuries, particularly during the early rehabilitation phase. The progressive increase in daily activity may reflect recovery of mobility and functional independence during the early rehabilitation phase. However, the present data do not allow conclusions regarding standardized recovery thresholds or normative activity trajectories. Where available, pre-injury step count was documented to contextualize postoperative trajectories. Descriptively, the plotted mean curves suggest that patients with upper extremity injuries approached their pre-injury activity levels earlier, whereas recovery of step count was slower in patients with lower extremity injuries. However, because baseline step count data were available only for a subset of participants, these descriptive comparisons should be interpreted cautiously and selection bias cannot be excluded.

In upper extremity injuries, step count changes likely reflect overall activity behavior rather than limb-specific functional recovery. In this context, wearable inertial motion analysis may provide complementary information on movement quality and upper-limb function that cannot be captured by step count alone [30]. Our observations regarding postoperative activity patterns are consistent with previous findings and with relevant literature investigating wearable-based activity monitoring in musculoskeletal injuries. Several studies have demonstrated that daily step count decreases significantly after lower extremity fractures and gradually increases during the rehabilitation phase [21,23,31]. The descriptive analysis of the time point at which patients first achieved 5000 steps per day further illustrates these differences between injury locations. The data show that the vast majority (95%) of patients with upper extremity injuries were able to reach this threshold within the first three months. In contrast, only about three-quarters (73%) of patients with lower extremity injuries achieved the same. The difference becomes even clearer when examining the average day on which this milestone was first reached: after approximately three weeks in upper extremity injuries, but only after more than eight weeks in lower extremity injuries. Given the absence of an evidence-based definition of a clinically meaningful step count threshold, this value should be interpreted as a pragmatic reference rather than a marker of recovery or health. Reaching this threshold on a single day should not be interpreted as evidence of sustained functional recovery.

The observed differences in postoperative step count were also associated with patient-reported health status and return-to-work outcomes. Associations between postoperative step count and patient-reported outcomes have also been reported after total hip arthroplasty, where higher step counts were associated with better functional PROM scores during recovery [32]. In the present study, these associations were primarily observed in patients with lower extremity injuries during the early postoperative period. Given the exploratory design of this study and the potential influence of unmeasured confounders, these findings should not be interpreted as evidence of causal relationships or as validated predictive models. Rather, they should be considered hypothesis-generating observations that warrant further investigation in larger, injury-specific studies. Step count may therefore serve only as a general indicator of activity behavior rather than a comprehensive measure of recovery. Combining step count data with complementary outcome measures, such as PROMs, motion analysis systems (camera- or inertial sensor-based), or implantable sensors, may provide a more comprehensive assessment of postoperative recovery. The potential value of considering complementary measures is illustrated by findings after anterior cruciate ligament reconstruction, where daily step counts were associated with differences in gait biomechanics and joint loading patterns [33]. In this context, wearable sensor-based approaches, particularly those using inertial measurement units, have increasingly been applied to characterize gait events and pathological gait beyond conventional activity measures [34]. More broadly, studies in the field of wearable-derived digital motor biomarkers have demonstrated that IMU-based gait features, including approaches combined with machine-learning techniques, can provide objective information on movement quality and gait stability beyond activity volume alone [35,36]. Although neurological disorders differ fundamentally from orthopedic trauma, these developments illustrate the potential of complementary wearable-derived measures to provide information beyond simple step counts.

A higher proportion of patients with upper extremity injuries regained work ability within the first three months (89% vs. 49%). However, return to work is a multifactorial outcome that depends not only on postoperative mobility but also on fracture characteristics, treatment strategy, rehabilitation protocols, occupational demands, workplace accommodations, employment status, and individual socioeconomic factors. As these variables were not systematically recorded in the present study, the observed differences should not be interpreted as evidence that postoperative step count can independently determine return-to-work status. Rather, step count may represent one component of the overall recovery process and should be interpreted as a correlate, rather than an independent determinant, of return-to-work outcomes.

A major strength of this study is its prospective real-world design, which enabled continuous monitoring of postoperative activity under everyday conditions. The prospective collection of wearable-derived activity data was complemented by retrospectively supplemented clinical parameters. The use of participants’ personal wearable devices enabled continuous monitoring of physical activity while minimizing recall and observer bias. At the same time, this pragmatic real-world approach inevitably reduced methodological standardization because device characteristics other than device manufacturer and wear conditions were not controlled. These results support the growing trend toward integrating wearable technology as a complementary monitoring tool in orthopedics and trauma surgery [14]. The present findings further suggest that such digital mobility measures should be interpreted within their specific clinical context and alongside complementary outcome measures rather than as stand-alone indicators of recovery.

From a clinical and research perspective, the present findings support the use of step count as a pragmatic baseline parameter to describe ambulatory activity after surgery, particularly after lower extremity fractures where mobility restrictions are directly reflected in step count trajectories. In this setting, step count trends may complement routine follow-up by providing an activity-based description of recovery under everyday conditions. In upper extremity fractures, step count should always be interpreted alongside complementary outcome measures. More broadly, there is increasing interest in using step count as a baseline parameter in orthopaedics, especially in lower extremity injuries and degenerative conditions [37]. At the same time, the full value of activity monitoring often emerges when step count is combined with additional, entity-specific outcomes [38]. In this context, the present study sharpens the understanding of what step count can contribute when used in isolation and where additional parameters are needed. Looking ahead, step count is likely to be used more frequently in follow-up due to its low burden and broad availability, while efforts continue to complement it with more specific outcome measures.

Several methodological limitations should be acknowledged. Participants used different consumer wearable devices without standardized documentation of software version, wear location, or daily wear duration. Missing wearable observations occurred under real-world conditions and were not imputed. Consequently, measurement variability and injury-specific measurement bias cannot be excluded, particularly because reduced arm swing after upper extremity injuries may affect step detection in wrist-worn devices. Step count reflects ambulatory activity but not limb-specific function, pain, strength, coordination, or gait quality and may be influenced by walking aids. The study was exploratory and non-randomized. Longitudinal step count trajectories were analyzed descriptively and no formal repeated-measures models were applied. Therefore, the observed trajectories should be interpreted as descriptive patterns rather than inferential estimates of recovery. Furthermore, important clinical and occupational variables, including fracture severity, postoperative weight-bearing restrictions, rehabilitation protocols, occupational demands, employment characteristics, and workplace accommodations, were not systematically recorded in this exploratory analysis. Residual confounding therefore cannot be excluded, particularly with regard to the analyses of return to work. The follow-up period was limited to three months, and pre-injury step count data were available only for a subset of participants. Consequently, descriptive comparisons with baseline activity should be interpreted cautiously because selection bias cannot be excluded. Finally, inclusion was limited to patients who already used consumer wearable devices before injury. This may have introduced selection bias toward a more technology-affine and potentially more active population, thereby limiting generalizability to the overall trauma population.

These results provide preliminary insights into step count as an activity-based outcome in orthopedic trauma. It is a pragmatic measure of mobility, particularly in lower extremity injuries, but limited for assessing upper extremity functional recovery. Further studies should incorporate standardized wearable documentation, predefined wear-time criteria, longitudinal statistical modeling, and detailed clinical and occupational variables together with complementary digital mobility outcomes to further define the role of step count in orthopedic trauma recovery.

## 5. Conclusions

In this exploratory cohort, postoperative step count trajectories differed between upper and lower extremity fractures, with lower extremity injuries showing markedly lower activity during the early postoperative recovery period. Step counts increased over time in both groups, and the descriptive 5000-step activity milestone was reached earlier in the upper extremity group than in the lower extremity group. In upper extremity injuries, step count should be interpreted with caution because it reflects overall activity behavior rather than limb-specific functional recovery. Given the exploratory design, the heterogeneous real-world use of consumer wearable devices, and the potential for residual confounding, including unmeasured occupational and treatment-related factors, these findings should be considered preliminary and hypothesis-generating. Future studies using standardized wearable documentation and complementary digital mobility outcomes are needed to further evaluate the role of step count in orthopedic trauma recovery across different fracture entities.

## Data Availability

All original data are provided in this publication. No additional datasets are available.

## Abbreviations

The following abbreviations are used in this manuscript:

PROMs	Patient-reported outcome measures
PROMIS	Patient-Reported Outcomes Measurement Information System
NRS	Numerical Rating Scales

## References

[1] Grifka J. (2021). Orthopädie Unfallchirurgie.

[2] Vogel C., Haas D., Histing T., Braun B.J. (2025). Osteosynthesis of subtrochanteric femoral fractures in adults. Chirurgie.

[3] Vogel C., Reumann M.K., Menger M.M., Herath S.C., Rollmann M.F.R., Lauer H., Histing T., Braun B.J. (2024). Non-unions of the upper extremities. Chirurgie.

[4] Bücking B., Neuerburg C., Knobe M., Liener U. (2019). Treatment of patients with fragility fractures. Unfallchirurg.

[5] Grogan Moore M.L., Jayakumar P., Laverty D., Hill A.D., Koenig K.M. (2019). Patient-Reported Outcome Measures and Patient Activation: What Are Their Roles in Orthopedic Trauma?. J. Orthop. Trauma.

[6] Gregory J.J., Werth P.M., Reilly C.A., Lucas A.P., Bessen S.Y., Rode J.B., Morrissey J.A., Sparks M.B., Jevsevar D.S., Gitajn I.L. (2022). Trajectory of Recovery in PROMIS Global Physical Health After Surgical Fracture Fixation. J. Am. Acad. Orthop. Surg..

[7] Vogel C., Grimm B., Marmor M.T., Sivananthan S., Richter P.H., Yarboro S., Hanflik A.M., Histing T., Braun B.J. (2024). Wearable Sensors in Other Medical Domains with Application Potential for Orthopedic Trauma Surgery-A Narrative Review. J. Clin. Med..

[8] Ladha K.S., Lu J., McIsaac D.I., van Vlymen J.M., Lebovic G., Ehtesham S., Pazmino-Canizares J., Clarke H., Parotto M., Lorello G.R. (2023). Peri-Operative Wearables in Elder Recover after Surgery (POWERS) study: A protocol for a multicentre, prospective cohort study to evaluate perioperative activity with postoperative disability in older adults after non-cardiac surgery. BMJ Open.

[9] Lind C.M., Abtahi F., Forsman M. (2023). Wearable Motion Capture Devices for the Prevention of Work-Related Musculoskeletal Disorders in Ergonomics-An Overview of Current Applications, Challenges, and Future Opportunities. Sensors.

[10] Yanagisawa T., Tatematsu N., Horiuchi M., Migitaka S., Yasuda S., Itatsu K., Kubota T., Sugiura H. (2022). Preoperative physical activity predicts postoperative functional recovery in gastrointestinal cancer patients. Disabil. Rehabil..

[11] Braun B.J., Grimm B., Hanflik A.M., Richter P.H., Sivananthan S., Yarboro S.R., Marmor M.T. (2022). Wearable technology in orthopedic trauma surgery—An AO trauma survey and review of current and future applications. Injury.

[12] Győrffy Z., Boros J., Döbrössy B., Girasek E. (2023). Older adults in the digital health era: Insights on the digital health related knowledge, habits and attitudes of the 65 year and older population. BMC Geriatr..

[13] Braun B.J., Hofmann K., Meierhofer C.N., Menger M.M., Maisenbacher T.C., Vogel C., Haas D., Marmor M.T., Histing T., Braun E.-M. (2025). Patient Recruitment Characteristics for Wearable-Sensor-Based Outcome Assessment in Trauma Surgery. J. Clin. Med..

[14] Braun B.J., Histing T., Menger M.M., Platte J., Grimm B., Hanflik A.M., Richter P.H., Sivananthan S., Yarboro S.R., Gueorguiev B. (2023). “Bring Your Own Device”—A New Approach to Wearable Outcome Assessment in Trauma. Medicina.

[15] Braun B.J., Osche D., Rollmann M., Orth M., Mörsdorf P., Histing T., Pohlemann T., Herath S.C. (2019). Increased therapy demand and impending loss of previous residence status after proximal femur fractures can be determined by continuous gait analysis—A clinical feasibility study. Injury.

[16] Hameed D., Sodhi N., Dubin J., Schneider A., Barrack R.L., Mont M.A. (2024). Integrating Smartphone Applications and Wearable Devices for Postoperative Rehabilitation in Total Knee Arthroplasty: A Critical Review. J. Arthroplast..

[17] Konnyu K.J., Thoma L.M., Cao W., Aaron R.K., Panagiotou O.A., Bhuma M.R., Adam G.P., Pinto D., Balk E.M. (2023). Prehabilitation for Total Knee or Total Hip Arthroplasty: A Systematic Review. Am. J. Phys. Med. Rehabil..

[18] Su W., Zhou Y., Qiu H., Wu H. (2022). The effects of preoperative rehabilitation on pain and functional outcome after total knee arthroplasty: A meta-analysis of randomized controlled trials. J. Orthop. Surg. Res..

[19] Constantinescu D., Pavlis W., Rizzo M., Vanden Berge D., Barnhill S., Hernandez V.H. (2022). The role of commercially available smartphone apps and wearable devices in monitoring patients after total knee arthroplasty: A systematic review. EFORT Open Rev..

[20] Polhemus A., Delgado-Ortiz L., Brittain G., Chynkiamis N., Salis F., Gaßner H., Gross M., Kirk C., Rossanigo R., Taraldsen K. (2021). Walking on common ground: A cross-disciplinary scoping review on the clinical utility of digital mobility outcomes. npj Digit Med..

[21] Marmor M.T., Grimm B., Hanflik A.M., Richter P.H., Sivananthan S., Yarboro S.R., Braun B.J. (2022). Use of Wearable Technology to Measure Activity in Orthopaedic Trauma Patients: A Systematic Review. Indian J. Orthop..

[22] Kammerlander C., Blauth M., Gosch M., Böcker W. (2015). Co-management in geriatric traumatology. Orthopade.

[23] Haas D., Hofmann K., Meierhofer C., Menger M.M., Maisenbacher T., Vogel C., Histing T., Braun B.J. (2025). First Results of a Prospective Cohort Study Assessing the Applicability of Step Counts as a Digital Mobility Outcome in Trauma Surgery—A Brief Report. Z. Orthop. Unfallchirurgie.

[24] Shear B.M., Brodke D.J., Hancock G.R., McGlone P., Demyanovich H., Li V., Bell A., Okhuereigbe D., Slobogean G.P., O’Toole R.V. (2025). Predicting Post-Fracture Recovery with Smartphone Mobility Data: A Proof-of-Concept Study. J. Bone Jt. Surg. Am..

[25] Sluys K.P., Shults J., Richmond T.S. (2016). Health related quality of life and return to work after minor extremity injuries: A longitudinal study comparing upper versus lower extremity injuries. Injury.

[26] Simonsick E.M., Kasper J.D., Guralnik J.M., Bandeen-Roche K., Ferrucci L., Hirsch R., Leveille S., Rantanen T., Fried L.P. (2001). Severity of upper and lower extremity functional limitation: Scale development and validation with self-report and performance-based measures of physical function. J. Gerontol. Ser. B Psychol. Sci. Soc. Sci..

[27] Fuller D., Colwell E., Low J., Orychock K., Tobin M.A., Simango B., Buote R., Van Heerden D., Luan H., Cullen K. (2020). Reliability and Validity of Commercially Available Wearable Devices for Measuring Steps, Energy Expenditure, and Heart Rate: Systematic Review. JMIR mHealth uHealth.

[28] Evenson K.R., Goto M.M., Furberg R.D. (2015). Systematic review of the validity and reliability of consumer-wearable activity trackers. Int. J. Behav. Nutr. Phys. Act..

[29] Shei R.J., Holder I.G., Oumsang A.S., Paris B.A., Paris H.L. (2022). Wearable activity trackers-advanced technology or advanced marketing?. Eur. J. Appl. Physiol..

[30] Ippolito G., Damo M., Ferraro S., Trabassi D., Serrao M., Lanzetti R.M., Surace M.F., Mazza D. (2026). Pilot biomechanical study of complex upper-limb movements in patients with RSA using inertial sensors: Feasibility of sport-specific gestures. Shoulder Elb..

[31] Daskivich T.J., Houman J., Lopez M., Luu M., Fleshner P., Zaghiyan K.N., Cunneen S., Burch M., Walsh C.A., Paiement G. (2019). Association of Wearable Activity Monitors With Assessment of Daily Ambulation and Length of Stay Among Patients Undergoing Major Surgery. JAMA Netw. Open.

[32] Orsi A.D., Mathew M., Plaskos C., Wakelin E.A., Slotkin E.M., Coffey S., Ponder C.E., Keggi J.K., McMahon S.J. (2024). Patient reported outcome measures correlate with step-count in total hip arthroplasty. Technol. Health Care.

[33] Lisee C., Davis-Wilson H.C., Evans-Pickett A., Horton W.Z., Blackburn J.T., Franz J.R., Thoma L.M., Spang J.T., Pietrosimone B. (2022). Linking Gait Biomechanics and Daily Steps After ACL Reconstruction. Med. Sci. Sports Exerc.

[34] Prasanth H., Caban M., Keller U., Courtine G., Ijspeert A., Vallery H., von Zitzewitz J. (2021). Wearable Sensor-Based Real-Time Gait Detection: A Systematic Review. Sensors.

[35] Trabassi D., Serrao M., Varrecchia T., Ranavolo A., Coppola G., De Icco R., Tassorelli C., Castiglia S.F. (2022). Machine Learning Approach to Support the Detection of Parkinson’s Disease in IMU-Based Gait Analysis. Sensors.

[36] Castiglia S.F., Tatarelli A., Trabassi D., De Icco R., Grillo V., Ranavolo A., Varrecchia T., Magnifica F., Di Lenola D., Coppola G. (2021). Ability of a Set of Trunk Inertial Indexes of Gait to Identify Gait Instability and Recurrent Fallers in Parkinson’s Disease. Sensors.

[37] Braun B.J., Grimm B., Marmor M.T., Osterhoff G., Back D.A., Menger M.M., Vogel C., Histing T., Haas D., AO Trauma Digital Expert Group (2026). Step count as a digital mobility outcome in orthopedics and orthopedic trauma surgery: A scoping review. EFORT Open Rev..

[38] Braun B.J., Menger M.M., Histing T., Marmor M.T., Grimm B., Fischer F., Harrison C., Joeris A., Lambert S., AO Upper Extremity Global Expert Committee (2025). Towards a comprehensive digital wearable tracking system of the patient recovery journey after extremity trauma: A narrative review. EFORT Open Rev..

